# Malnutrition as an independent risk factor for incident delirium in cohort of older adults receiving domiciliary care services

**DOI:** 10.1007/s11357-025-02040-7

**Published:** 2025-12-11

**Authors:** Benedicte Huseby Bøhn, Maria Krogseth, Nina Jøranson, Torgeir Bruun Wyller, Geir Selbæk, Gro Gujord Tangen

**Affiliations:** 1 The Norwegian National Centre for Ageing and Health, Vestfold Hospital Trust, Tønsberg, Norway; 2 Department of Geriatric Medicine, Oslo University Hospital, Oslo, Norway; 3 Oslo Delirium Research Group, Department of Geriatric Medicine, Oslo University Hospital, Oslo, Norway; 4 Old Age Psychiatry Research Network, Telemark Hospital Trust and Vestfold Hospital Trust, Tønsberg, Norway; 5 Department of Internal Medicine, Vestfold Hospital Trust, Tønsberg, Norway; 6 Faculty of Health and Social Sciences, Department of Nursing and Health Science, University of South-Eastern Norway, Drammen, Norway; 7 Faculty of Health Sciences, VID Specialized University, Oslo, Norway; 8 Institute of Clinical Medicine, Faculty of Medicine, University of Oslo, Oslo, Norway

**Keywords:** Nutritional status, Acute confusional state, Risk factors, Frailty, The primary healthcare service

## Abstract

**Background:**

Malnutrition and frailty frequently affect older adults receiving domiciliary care services, increasing their vulnerability to adverse events such as delirium. Despite this, the role of malnutrition as a risk factor for delirium in this population remains under-researched. The aim of this study was to examine the relationship between nutritional status and the development of delirium over a 2-year period among older adults who received domiciliary care services.

**Method:**

In this 2-year prospective cohort study, we included 210 participants aged 65 years or older who received domiciliary care services at least once per week. Nutritional status was assessed using the Mini Nutritional Assessment at the time of inclusion, while delirium was assessed weekly, upon admission to hospital, and upon clinical indication according to DSM-5 criteria. Logistic regression analysis was used to examine the relationship between malnutrition and delirium.

**Results:**

Of the sample, 116 (55.2%) were malnourished/at risk of malnutrition at the time of inclusion. Over a 2-year period, 42.4% developed delirium. The odds ratio for developing delirium was 2.00 (95% CI 1.08–3.72, *P* = 0.028), for the group with malnutrition/risk of malnutrition, adjusted for covariates.

**Conclusion:**

Malnutrition is an independent risk factor for delirium in older adults receiving domiciliary care services. These findings highlight the importance of regular nutritional assessments and interventions to potentially reduce the risk of delirium in this vulnerable population.

## Introduction

Delirium, a complex neuropsychiatric syndrome characterised by disturbed arousal, attention, and cognition, is most often triggered by an acute somatic illness or trauma [1]. While delirium can occur in young and robust individuals if they experience severe illness or trauma, older adults living with frailty are far more likely to experience delirium even in response to minor stressors [2]. Delirium is a significant burden for both patients and relatives, and the prevention and managing of delirium is a demanding challenge for healthcare professionals at all levels of the healthcare system [3–5]. While delirium is well studied in hospital settings, only three (1%) of the included studies in a recent review of predisposing factors for delirium were conducted among community-dwelling older adults [6]. This comprehensive review by Ormseth et al. [6] included over 300 studies, and among the many predisposing and precipitating factors associated with delirium were advanced age, cognitive impairment, functional impairment (including frailty), and comorbidity. While these factors are not easily modifiable, the authors also identified malnutrition as a possible predisposing factor for delirium [6]. Malnutrition, defined as a condition in which a lack of energy or protein leads to weight loss and reduced muscle mass, can lead to a decline in physical or mental function and worse disease outcomes [7]. Further, malnutrition is increasingly common with old age, more common in women than in men, and highly prevalent in recipients of domiciliary care services [8, 9]. Malnutrition is associated with many of the same negative outcomes as delirium, such as increased morbidity and mortality [10]. Other negative consequences of malnutrition include increased care needs, prolonged hospital stays, and higher readmission rates, placing a greater burden on caregivers and healthcare services [11, 12].

There is stronger evidence for preventing delirium than treating it. A meta-analysis concludes that almost half of all cases of delirium can be prevented with simple, but staff-intensive, non-pharmacological interventions, such as focusing on nutrition and hydration, mobilisation, cognitive stimulation, compensating for sensory deficits, and good sleep hygiene [13]. These multicomponent interventions have primarily been conducted in hospital or postoperative settings, where nutrition has been included as one of several preventive measures. However, little is known about the independent association between baseline nutritional status and the subsequent development of delirium among community-dwelling older adults receiving domiciliary care services. Understanding this relationship in a stable, non-acute population is important for identifying potentially modifiable risk factors that may inform preventive strategies beyond institutional care settings. Evidence for pharmacological treatment of delirium remains limited [14].

Based on the findings mentioned above, there is a need to establish a better understanding of the factors associated with delirium in stable conditions. The aim of this study was to assess whether nutritional status in a stable state was associated with the development of delirium. We used a cohort of older adults receiving weekly domiciliary care in a Norwegian municipality, performing validated delirium assessments both at home and during hospital admissions, over a 2-year follow-up period.

## Methods

### Study design and participants

The Capturing Acute and Social Care in Dependent Elders (CASCADE) study was a prospective cohort study, conducted in Sandefjord municipality in Norway [15]. The study design included a baseline home visit, followed by visits every 6 months for two years. Inclusion criteria were being over 65 years old and receiving domiciliary care at least once a week between May 2015 and July 2016. Exclusion criteria were expected life expectancy of less than two weeks, known Lewy body dementia, chronic disease that required help from domiciliary care from before the age of 65, or that the need for domiciliary care was due to substance abuse or severe psychiatric illness other than dementia. Participants with Lewy body dementia were excluded because the main clinical features of this disorder overlap with delirium, making it difficult to identify delirium in people with this dementia disease [16]. Eligible participants were identified by the manager of the domiciliary care team. In alphabetical order, the patients were consecutively offered participation by an employee in the domiciliary care team. Those providing oral consent received a home visit from a member of the research team, consisting of a doctor and two research nurses trained in geriatrics. Written informed consent was obtained by the research team at the first home visit. All participants were examined by the research team at baseline to ensure that they were in a stable clinical phase. Delirium was systematically assessed at inclusion using the Diagnostic and Statistical Manual of Mental Disorders (DSM-5) criteria, and no participant fulfilled the diagnostic criteria for delirium at baseline. Participants were included only when stable and free from acute illness or delirium. In cases where individuals had impaired capacity, consent was also obtained from a next-of-kin. The CASCADE study was approved by the Regional Committee for Medical and Health Research Ethics, REK (no. 2014/1972).

### Measures

#### Delirium assessments

The delirium diagnosis was assessed based on the DSM-5 criteria [1]. Each participant was screened for delirium weekly by members of the domiciliary care team using the Single Question in Delirium (SQiD): “Do you think *the patient’s name* has been more confused lately?” [17]. If the SQiD screening was positive, if there was an acute change in mental status, or if the participant was admitted to hospital, the project leader (a physician trained in geriatrics) carried out formal delirium diagnostics based on DSM-5 criteria. During hospital admissions, the participant was assessed daily until either delirium occurred, or the participant was considered stable. Delirium assessments were made using several validated tests for changes in the level of consciousness, orientation, attention, etc. For a detailed description of the tests and the implementation of the delirium diagnostics, see Krogseth et al. [15]. Based on all assessments during the study period, we created a dichotomous delirium variable (delirium or no delirium) for this study.

#### Nutritional status

The Mini Nutritional Assessment (MNA) was used to assess nutritional status. The MNA consists of 18 elements: anthropological measurements (weight, height, weight loss, arm and leg circumference), general status (lifestyle, medication use, and mobility), eating habits (number of meals, food and drink intake, and autonomy for food intake), and subjective assessment (self-perception of health and nutritional status) [18]. The total overall score (0–30 points) on the MNA was used as a measure of nutritional status, where < 17 points indicate malnutrition, 17–23.5 points risk of malnutrition, and 24–30 points normal nutritional status [19, 20]. In the analyses, the MNA was dichotomised to malnutrition/risk of malnutrition (≤ 23.5) vs. normal nutritional status (24–30).

#### Covariates

The Barthel ADL Index was used to measure independence in activities of daily living (ADL), such as meals, personal care, and mobility. The index is scored 0–20 where lower scores indicate less independence [21]. Comorbidity was assessed by Charlson’s comorbidity index, where a higher total score indicates more severe comorbidity [22]. Cognitive function was assessed using the Montreal Cognitive Assessment (MoCA), a multidomain cognitive screening instrument, scored from 0 to 30 where higher scores indicate better cognitive function [23]. Frailty status was described using a 34-item Frailty Index, as described in detail previously [24]. Demographic data were obtained from the patient, relatives, and the patient record from the domiciliary care team, see Table 1.

**Table 1:** Baseline characteristics by delirium status during the 2-year follow-up

	Whole sample ***N*** = 210	Delirium ***n*** = 89 (42.4%)	No delirium ***n*** = 121 (57.6%)	***p***
Age, mean (SD)	84.5 (8.3)	85.6 (7.6)	83.6 (8.7)	0.09ᵃ
Female, *n *(%)	138 (65.7%)	60 (67.4%)	78 (64.5%)	0.66ᵇ
Living alone, *n* (%)	151 (71.9%)	60 (67.4%)	91 (75.2%)	0.28ᵇ
Years of education, median (IQR)	9.0 (5.0)	9.0 (5)	8 (4)	0.15ᶜ
Barthel ADL index, median (IQR)	17.0 (4.0)	16.0 (4.0)	17.0 (4.0)	0.15ᶜ
Charlson Comorbidity Index, mean (SD)	2.6 (2.0)	3.0 (2.1)	2.4 (1.9)	0.03ᵃ
MoCA, mean (SD)	17.4 (6.5)	16.3 (6.7)	18.3 (6.2)	0.03^a^
BMI, mean (SD)^1^	25.2 (4.8)	24.3 (4.4)	25.9 (4.9)	0.02ᵃ
Frailty Index, mean (SD)	0.39 (0.1)	0.42 (0.1)	0.36 (0.1)	< 0.001^a^
MNA-total score, median (IQR)	23.0 (5.5)	22.0 (6.5)	24.0 (5.5)	0.01ᶜ
Malnourished (at risk of and malnutrition), *n* (%)	116 (55.2%)	59 (66.3%)	57 (47.1%)	0.008ᵇ
Graded nutritional status MNA, *n* (%)				0.006ᵇ
Normal nutritional status	94 (44.8%)	30 (33.7%)	64 (52.9%)	
At risk of malnutrition	93 (44.3%)	44 (49.4%)	49 (40.5%)	
Malnourished	23 (11.0%)	15 (16.9%)	8 (6.6%)	

*SD,* standard deviation; *IQR*, interquartile range; *MNA*, Mini Nutritional Assessment

ᵃIndependent samples *t*-test, ᵇchi-square test, ᶜMann-Whitney *U* test. ^1^ BMI missing = 7

### Statistical analyses

The association between nutritional status and delirium was investigated using logistic regression analysis, with delirium as the dependent variable and MNA as the exposure variable. We included age, sex, education, living situation, Barthel ADL index, Charlson’s comorbidity index, and MoCA as potentially confounding covariates based on clinical reasoning and previous studies [6]. Correlations between the variables in the model were investigated to check for multicollinearity (defined as correlation coefficients > 0.7).

Logistic regression analysis was carried out in two steps, and two different models are presented. In model 1, nutritional status, sex, and age were included. In model 2, Barthel ADL index, education, living alone, Charlson’s comorbidity index, and MoCA were additionally included. The association between the outcome variable and independent variables is reported as the odds ratio (OR) with 95% confidence interval (CI). The MNA was dichotomised into ≤ 23.5 (at risk of or malnourished) and > 23.5 (normal nutritional status). In addition, a sensitivity analysis was performed using logistic regression analysis, where the MNA was recoded into > 17 (normal nutritional status and at risk of malnutrition) versus ≤ 17 (malnutrition).

During the 2-year observation period, 63 (30%) of the participants died. Of these, 60 (95%) had one or more episodes of delirium, and three (5%) had no episodes. Six participants did not complete the study. All these participants were retained in the analyses with data from inclusion and until death or dropout from the study.

Statistical analyses were performed using the Statistical Package for the Social Sciences (SPSS) version 28, and the significance level was set at *p* < 0.05.

## Results

Table 1 describes the sample at baseline, which consisted of 210 people, of which 138 (65.7%) were women. The average age was 84.5 years (SD 8.3), range 65–102 years. According to the MNA scoring, 11.0% were malnourished and 44.3% were at risk of malnutrition. Over the 2-year follow-up period, 89 (42.4%) participants were diagnosed with one or more episodes of delirium. Of those who were malnourished or at risk for malnutrition, 59 (66.3%) developed delirium during the observation period. In the total sample, 201 (95.7%) of the participants were categorised as frail. The mean (SD) Frailty Index score was significantly higher in the group who developed delirium (0.42 [0.1]) compared to the group without delirium (0.36 [0.1]).

Table 2 shows the association between nutritional status and the risk of delirium. The variables used in the logistic regression analysis were nutritional status, sex, age, ADL, education, living alone, comorbidity, and MoCA. Model 1 shows that nutritional status (including age and sex) was associated with the development of delirium. The odds of developing delirium was 2.18 times higher (95% CI 1.23–3.85) for those with malnutrition or at risk of malnutrition, compared to those with normal nutritional status (*p* = 0.007). In model 2, controlled for other variables, the odds ratio (OR) for nutritional status was lower, OR = 2.00 (95% CI 1.08–3.72), but remained statistically significant (*p* = 0.028). In this model, Charlson’s comorbidity index was also significantly associated with delirium. The Nagelkerke R Square for the fully adjusted model was 0.15, indicating that the model explains 15% of the variation in delirium occurrence.

**Table 2:** Logistic regression analyses with incident delirium as dependent variable and Mini Nutritional Assessment as exposure variable

	Model 1 (n = 210)			Model 2 (n = 203)		
	OR	95% CI	***p***	OR	95% CI	***p***
**MNA**	2.18	1.23–3.85	0.007	2.00	1.08–3.72	0.028
**Sex**	1.02	0.55–1.89	0.94	1.58	0.79–3.18	0.195
**Age, years**	1.03	0.99–1.07	0.13	1.04	0.999–1.08	0.057
**Education, years**				1.08	0.98–1.19	0.11
**Barthel ADL**				0.99	0.91–1.08	0.85
**Living status**				1.46	0.74–2.87	0.26
**Charlson comorbidity index**				1.24	1.05–1.46	0.01
**MoCA, score**				0.96	0.92–1.01	0.14

*MNA* = Mini Nutritional Assessment, *ADL* = activities of daily living, *MoCA* = Montreal Cognitive Assessment. Model 1 is adjusted for age and sex, model 2 is adjusted for age, sex, education, Barthel ADL, living status, Charlson comorbidity index and MoCA score. *MNA*: 0 = normal nutritional status, 1 = malnutrition/risk of malnutrition; sex: 0=men, 1= women; living status: 0= living alone, 1= cohabiting

In the sensitivity analysis, where nutritional status was dichotomised as malnutrition versus at risk of malnutrition/normal nutritional status, the results remained largely unchanged. In the fully adjusted model, participants with malnutrition had an OR = 2.95 (95% CI 1.07–8.17), *p* = 0.037 of developing delirium.

## Discussion

In this longitudinal cohort study of 210 community-dwelling older adults receiving domiciliary care services, we found that being at risk of or having malnutrition was independently associated with the incidence of delirium during the 2-year follow-up. Our findings also indicate that advanced age and higher comorbidity are associated with an elevated risk of delirium. This study is unique in its comprehensive approach, as delirium assessments were conducted both at home and during hospital admissions using validated tests for delirium, ensuring rigorous evaluation with minimal loss to follow-up. Importantly, all participants were examined for delirium at baseline, and none met DSM-5 criteria for delirium at inclusion. The study therefore specifically captured incident delirium developing after the baseline assessment, ensuring that the observed associations reflect delirium occurring subsequent to the recorded nutritional status.

We have not identified any previous studies that explicitly examined the association between malnutrition and delirium in community-dwelling older adults receiving domiciliary care. Our results align with hospital-based research indicating that malnourished older adults have an increased risk of delirium [25–29]. However, these studies have primarily focused on postoperative delirium, where malnutrition has been linked to poorer surgical outcomes and an increased risk of delirium. Our results extend these findings by demonstrating that malnutrition is also an independent risk factor for developing delirium in older adults receiving domiciliary care. In our sample, a greater proportion of participants were at risk of malnutrition rather than having established malnutrition. The association between nutritional status and delirium remained significant in the sensitivity analysis when participants with established malnutrition were analysed separately, supporting the robustness of our findings. However, while the odds ratio increased in this subgroup, it was accompanied by an even wider confidence interval, indicating greater uncertainty around the effect estimate. This may suggest that more severe malnutrition could further elevate the risk of delirium, but the precision of this estimate is limited, and confirmation in larger studies is needed to clarify its clinical relevance.

Consistent with previous research, our study confirms that advanced age and a higher Charlson’s Comorbidity Index score are significant risk factors for delirium [6]. Cognitive impairment is also a well-established risk factor [30], but the association with MoCA score was not statistically significant in our model. As the main objective of this study was to examine the association between nutritional status and delirium, covariates such as cognitive function were included to adjust for potential confounding rather than to estimate their independent effects. This lack of association could also be attributed to the overall level of frailty and multimorbidity present in our entire sample. Importantly, even after adjustment for age, comorbidity, and cognition, malnutrition remained significantly associated with delirium, supporting its role as an independent risk factor.

Beyond its role as a general health marker, nutritional status may also influence delirium risk through underlying biological mechanisms. Chronic low-grade inflammation, commonly observed in frailty and cognitive decline, may mediate the association between poor nutritional status and delirium risk [2]. Poor nutritional status has been associated with systemic inflammation and endothelial dysfunction, processes that may impair both vascular and brain health and contribute to delirium vulnerability [2, 31, 32]. Furthermore, nutrients with epigenetic activity, such as polyphenols, methyl-donor nutrients, and omega-3 fatty acids, have been shown to modulate cardiovascular function and reduce systemic inflammation [33]. Since better cardiovascular health has been associated with reduced delirium incidence [34], these findings point to a possible mechanistic link through which malnutrition could increase susceptibility to delirium. In line with this, dyslipidaemia and other metabolic disturbances have been associated with postoperative delirium [35], supporting the relevance of vascular health in delirium vulnerability. Recent findings also indicate that better dietary quality, particularly a higher intake of polyunsaturated-fat-rich foods and specific metabolites, may protect against delirium through anti-inflammatory and neuroprotective effects [36]. Taken together, current evidence suggests that inflammatory, metabolic, and epigenetic processes may link nutritional status, cardiovascular health, and delirium vulnerability, supporting nutrition as a potential modifiable target for prevention. While these pathways provide a biological rationale for the observed association, our findings are based on data from a prospective observational study and do not establish causality. We therefore suggest caution in inferring a direct causal relationship between malnutrition and delirium. Malnutrition reduces physiological reserves and serves as a marker of frailty [37], increasing susceptibility to delirium in the presence of acute stressors such as illness or injury. Additionally, both malnutrition and delirium have been linked to adverse outcomes, including higher mortality, prolonged hospital stays, and an increased likelihood of institutionalisation among older adults living at home [38, 39].

Given that nutritional status is a potentially modifiable risk factor, targeted interventions to improve it may help promote robustness and reduce the risk of delirium in frail older adults. International guidelines emphasise routine nutritional screening for older adults across all healthcare settings [40]. Frail older adults often experience multiple transitions between hospital and home, which can lead to gaps in nutritional care [41, 42]. Although our study did not investigate these transitions directly, poor coordination of nutritional care has been identified as a challenge for older adults with delirium [43]. Structured and individualised nutrition management in domiciliary care settings may help address these challenges and mitigate the risk of both malnutrition and delirium. Such interventions should be tailored to individual preferences, needs, and capacities, taking into account comorbidities, cognitive function, and overall frailty status. Since delirium prevention strategies involving hydration, nutrition, and mobilisation have proven effective in hospital settings [13], our findings suggest that similar interventions could benefit older adults in community settings as well.

However, it is also important to acknowledge that not all malnutrition is reversible. In frail older adults, especially those in the final phase of life, weight loss and reduced nutritional intake may reflect a decline in physiological reserves rather than a modifiable risk factor. One contributing factor is anorexia of aging, a phenomenon characterised by a progressive decline in appetite and food intake due to physiological, hormonal, and sensory changes associated with aging [44]. This age-related reduction in appetite can limit the effectiveness of intensive nutritional interventions. In such cases, intensive nutritional interventions may not be appropriate, and the focus should shift toward optimising comfort and quality of life. Considering both malnutrition and delirium as indicators of increased vulnerability, clinical decision-making should follow an individualised approach aligning with the person’s prognosis and care goals [10].

### Strengths and limitations

The study has some limitations that should be considered. The single-municipality setting may limit the generalizability of our findings to other regions or healthcare systems. Furthermore, the patients’ comorbidity may be more severe than recorded, as our information was based on registered diagnoses and reports from the patient and their relatives rather than on independent diagnostic assessments. Despite adjustment for important variables, the study was not powered to account for all potential confounders. Residual confounding from factors such as prior delirium, depression, medication use, and sensory impairments may remain, warranting cautious interpretation of the findings. Nevertheless, the relatively high prevalence of delirium likely supported adequate power to detect moderate associations, as reflected in the significant finding for nutritional status, although smaller effects may have gone undetected. Delirium assessments were conducted by a single physician who was not blinded to nutritional status, which may have introduced diagnostic bias. However, as clinical signs of established malnutrition are often readily apparent to a trained geriatrician, complete blinding may have been challenging. The diagnosis was nonetheless based on DSM-5 criteria and supported by multiple validated assessment tools. Further, although the SQiD has shown reasonable sensitivity (80%) for detecting delirium in older patients with cancer [17], its performance in frail older adults is not established, and some delirium cases may have been missed**.**

The strengths of the study include wide inclusion and few exclusion criteria, ensuring that participants are largely representative of the population. Participants were randomly selected for invitation, strengthening external validity. Data were collected using validated screening tools, and the study achieved complete data for the variables included in the analyses, along with a low dropout rate. Dropout analyses show that the participants who did not complete the study did not differ from the other participants [15]. The research team conducting the data collection consisted of only three professional health workers, helping to limit systematic bias.

## Conclusion

Our findings suggest that malnutrition is an independent risk factor for incident delirium among community-dwelling older adults receiving domiciliary care services. The absence of prior studies specifically investigating this population highlights a need for further research to confirm and extend our results. Meanwhile, our results underscore the importance of routine nutritional screening and targeted interventions to reduce the risk of delirium in this vulnerable group. Future studies should examine whether improving nutritional status can attenuate delirium risk.

## Data Availability

The full data set is, due to ethical restrictions, only available to the reader upon reasonable request to the project manager. Contact the corresponding author for contact information.
